# Genetic dissection of cell wall defects and the strigolactone pathway in Arabidopsis

**DOI:** 10.1002/pld3.149

**Published:** 2019-06-22

**Authors:** Vicente Ramírez, Markus Pauly

**Affiliations:** ^1^ Institute for Plant Cell Biology and Biotechnology and Cluster of Excellence on Plant Sciences (CEPLAS) Heinrich Heine University Düsseldorf Germany

**Keywords:** cell wall, cell wall integrity, cell wall sensing, *irregular xylem*, *O*‐acetylation, strigolactones

## Abstract

Defects in the biosynthesis and/or deposition of secondary plant cell wall polymers result in the collapse of xylem vessels causing a dwarfed plant stature and an altered plant architecture termed *irregular xylem* (*irx*) syndrome. For example, reduced xylan *O*‐acetylation causes strong developmental defects and increased freezing tolerance. Recently, we demonstrated that the *irx* syndrome in the *trichome birefringence‐like 29/eskimo1* (*tbl29/esk1*) mutant is dependent on MORE AXILLARY GROWTH 4 (MAX4), a key enzyme in the biosynthesis of the phytohormone strigolactone (SL). In this report, we show that other xylan‐ and cellulose‐deficient secondary wall mutants exhibit increased freezing tolerance correlated with the *irx* syndrome. In addition, these phenotypes are also dependent on MAX4, suggesting a more general interaction between secondary wall defects and SL biosynthesis. In contrast, MAX4 does not play a role in developmental defects triggered by primary wall deficiencies, suggesting that the interaction is restricted to vascular tissue. Through a reverse genetics approach, the requirement of different components of the SL pathway impacting the *irx* syndrome in *tbl29* was evaluated. Our results show that the *tbl29*‐associated *irx* phenotypes are dependent on the MAX3 and MAX4 enzymes, involved in the early steps of SL biosynthesis. In contrast, this signaling is independent on downstream enzymes in the biosynthesis and perception of SL such as MAX1 and MAX2.

## 1 INTRODUCTION

Xylem elements are surrounded by a secondary wall composed mainly of cellulose, xylan, and lignin, conferring unique physiochemical properties to these specialized cells. The functionality of the vascular system relies on the correct composition and structure of these modified walls, and most mutants affected in the biosynthesis and/or deposition of these three polymers show a collapsed vascular system (Brown, Zeef, Ellis, Goodacre, & Turner, 2005; Jones, Ennos, & Turner, 2001; Turner & Somerville, 1997). These so‐called *irregular xylem* (*irx*) mutants also exhibit severe growth defects including a dwarfed stature, reduced seed production, and an altered plant architecture. In addition, several studies show that these developmental problems—globally termed as *irregular xylem* syndrome—are often accompanied by constitutive activation of stress responses (Chen et al., 2005; Keppler & Showalter, 2010; Xin & Browse, 1998). For example, reduction of xylan *O*‐acetylation in *tbl29/esk1* mutant plants causes drastic xylem collapse accompanied by dwarfism and constitutive tolerance to freezing, drought, and salt stresses (Bouchabke‐Coussa et al., 2008; Lefebvre et al., 2011; Xin & Browse, 1998; Xin, Mandaokar, Chen, Last, & Browse, 2007; Xiong, Cheng, & Pauly, 2013). These phenotypes seem not to be a direct consequence of the defective secondary wall composition/structure, as two suppressor lines have been identified exhibiting rescued developmental‐ and stress‐related phenotypes while the low xylan *O*‐acetylation content was unchanged. In one case, *kaktus* (*kak*) mutant alleles suppress the *tbl29 irx* syndrome by boosting the vascular development resulting in normal xylem morphology and restoration of the water transport capacity (Bensussan et al., 2015). How *KAK,* a putative ubiquitin E3 ligase involved in endoreduplication and control of DNA content in trichomes, regulates xylem development remains elusive (Bensussan et al., 2015; Downes, Stupar, Gingerich, & Vierstra, 2003; El Refy et al., 2003). In another case, mutations in *MAX4* similarly rescue *tbl29* collapsed xylem, dwarfism, and increased freezing tolerance (Ramírez, Xiong, Mashiguchi, Yamaguchi, & Pauly, 2018). *MAX4* encodes a key enzyme in SL biosynthesis (Sorefan et al., 2003), suggesting that the *tbl29*‐triggered *irx* syndrome is SL‐dependent. This requirement was further confirmed by the chemical complementation assays using a synthetic SL (i.e., GR‐24). *tbl29 max4* double mutant plants growing in the presence of GR‐24 exhibit collapsed xylem and dwarfed stature, indicating that complementing the SL deficiency with exogenous applications of SL circumvent the *max4* effect (Ramírez et al., 2018).

SLs are a group of structurally similar compounds derived from carotenoids. Diverse SL compounds have been involved in the regulation of multiple processes in plants such as the interaction with root‐parasitic plants and symbiotic arbuscular mycorrhizal fungi; developmental processes, including photomorphogenesis, root architecture, senescence, flower development, or secondary growth; and adaptation responses to various biotic and abiotic stresses. However, the most extensively studied is the regulation of shoot branching (reviewed in Waters, Gutjahr, Bennett, & Nelson, 2017). The characterization of mutants showing a highly branched phenotype in Arabidopsis (*more axillary growth*;* max*), pea (*ramosus*;* rms*), rice (*high‐tillering dwarf/dwarf*;* htd/d*), and petunia (*decreased apical dominance*;* dad*) has been responsible for the identification of multiple components involved in the SL biosynthesis and signal transduction pathway (Arite et al., 2007; Booker et al., 2004; Johnson et al., 2006; Snowden et al., 2005; Sorefan et al., 2003; Zou et al., 2006). In the first step of the SL biosynthesis, a plastid‐localized β‐carotene isomerase (AtD27 in Arabidopsis) converts all‐*trans*‐β‐carotene into 9‐*cis*‐β‐carotene, which is then used as a substrate to generate carlactone by the consecutive action of two carotenoid cleavage dioxygenase (CCD) enzymes, CCD7/MAX3/RMS5/D17/DAD3 and CCD8/MAX4/RMS1/D10/DAD1 (Alder et al., 2012; Schwartz, Qin, & Loewen, 2004; Waters et al., 2012). Carlactone seems to be the first active SL compound in the pathway and is thought to act as the precursor for other SL compounds (Abe et al., 2014; Scaffidi et al., 2013; Seto et al., 2014). The production of carlactone and its following conversion into carlactonic acid (in Arabidopsis likely performed by the MAX1 cytochrome P450 enzyme) seem to be a common strategy in plants. In contrast, it seems that the pathway diverges from there, and species‐specific reactions transform carlactone into more than 20 canonical and non‐canonical SL compounds identified in plant exudates (Al‐Babili & Bouwmeester, 2015; Iseki et al., 2018; Tokunaga, Hayashi, & Akiyama, 2015). In Arabidopsis, carlactonic acid is transformed into methyl carlactonoate (MeCLA) by an unknown enzyme (Abe et al., 2014). MeCLA then binds and induces conformational changes to the D14 α/β‐fold hydrolase allowing the interaction with a SKP1‐CUL1‐F‐box‐protein (SCF)‐type ubiquitin ligase complex to transmit the SL signal via proteasomal degradation of negative regulators of SL signaling. An integral component of this complex, the MAX2 leucine rich F‐box protein, seems to be involved in the ubiquitination of putative transcriptional regulators controlling most of the SL‐dependent responses characterized so far, including members of the SUPPRESSOR OF MAX2 (SMAX1) and SMAX1‐LIKE families (Bennett et al., 2016; Jiang et al., 2013; Soundappan et al., 2015; Wang et al., 2015). However, the function and exact mechanism of action of most of these target proteins are still under debate. Upon D14‐MeCLA interaction, D14 returns to the initial conformation inducing the hydrolytic degradation of MeCLA and restoring the system after signal transmission (Seto et al., 2019).

## 2 MATERIALS AND METHODS

### 2.1 Growth conditions

Seeds were stratified in 0.2% Agarose for 4 days in the dark at 4°C. After stratification, seeds were sown on soil and grown in environmentally controlled growth chambers (8,000 luxes, 16‐hr light, 22°C/8‐hr dark, 19°C).

### 2.2 Mutant genotyping

Double mutants were generated by crossing and identified by genotyping of individuals in the F2 generation. Genotyping of T‐DNA lines was performed as described in Ramírez et al., 2018 (see Table S3 for primer sequences). To genotype *max2‐1* mutation, the amplicon generated by PCR using *max2‐1F/max2‐1 R* primer combination was digested with *ApoI* restriction enzyme resulting in a 147 bp single fragment in wildtype plants and two fragments (76 and71 bp) in mutant plants. *max3‐9* genotyping was performed using *max3‐9 F/max3‐9 R* primer combination, resulting in 120 bp PCR amplicon in wildtype plants and 131 bp in the mutant. To genotype the EMS‐derived *max1* and *max2‐2* mutations, PCR reactions with *max1F*/*max1 R* and *max2‐2F/max2‐2 R* primer combinations were performed and the respective amplicons were sequenced. Genotyping of prc1‐1 was done with *pcr1‐1F/pcr1‐1 R* primer combination and further digestion with *HpyCH4V* restriction enzyme resulting in three fragments in the wildtype (135, 70, and 56 bp) and only two in the mutant (135 and 126 bp). ixr2‐1 genotyping was done using the following primer combinations: *ixr2‐1* Fin/*ixr2‐1* Rout, resulting in amplification only in wildtype plants; *ixr2‐1* Fout/*ixr2‐1* Rin, resulting in amplification only in mutant plants; and *ixr2‐1* Fout/*ixr2‐1* Rout, resulting in amplification in all individuals. Primer sequences are described in Table S4.

The different mutant lines used in this study were obtained from the NASC (http://arabidopsis.info) (Scholl, May, & Ware, 2000) and ABRC (https://abrc.osu.edu) Arabidopsis stock centers. Stock numbers for each mutant line are described in Table S5.

### 2.3 Freezing experiments

Four‐week‐old plants of the indicated genotypes were incubated at −5°C in a Coolfreeze CF‐110 cooling box (Waeco) for 20 hr. After the freezing treatment, plants were transferred to the growth chamber. Survival rates were calculated as the % of plants alive after 3 days. Individual pictures were taken before and after the freezing exposure using a Lumix DMC‐FZ35 camera (Panasonic).

### 2.4 Plant measurements

Plant height and the number of axillary branches were recorded on 6‐week‐old plants. Individual pictures were taken using a Lumix DMC‐FZ35 camera (Panasonic).

Seedling length measurements were performed as described in Fagard et al., 2000 with minor modifications. Briefly, seeds were surface‐sterilized in a solution of 70% ethanol and 0.01% Tween‐20 and sown in half strength MS‐containing media without sucrose (Duchefa). Plates were incubated for 2 days at 4°C, then exposed to light for 1 hr to synchronize germination, covered in two layers of aluminum foil and incubated at 24°C for 4 days. Hypocotyls were horizontally transferred to agar plates and their image was recorded with a Lumix DMC‐FZ35 camera (Panasonic). Hypocotyl lengths were measured using ImageJ free software.

### 2.5 Xylem morphology

Xylem morphology was evaluated as described in Ramírez et al. (2018). Equivalent segments of plant stems from the various genotypes were used to prepare hand‐cut sections. After incubation in 0.02% Toluidine Blue O solution (Sigma‐Aldrich) for 2 min, sections were washed three times with sterile water and observed under a bright‐field lighting microscope (Leica DM2000 LED). At least 10 plants/genotype and 20 sections/plant were evaluated.

### 2.6 Cell wall composition

Primary stems from individual 6‐week‐old plants were cut into 1‐cm segments and ground for 2 min in a MM400 mixer mill (Retsch Technology) after lyophilization using a ScanVac CoolSafe Freeze‐dryer (Labogene). Total destarched alcohol‐insoluble residue was prepared and cellulose content, matrix polysaccharide composition, and total wall acetate were determined according to Foster, Martin, and Pauly (2010) as described in Ramírez et al. (2018).

## 3 RESULTS

### 3.1 Several cellulose‐ and xylan‐deficient mutants show increased freezing tolerance

**Figure 1:**
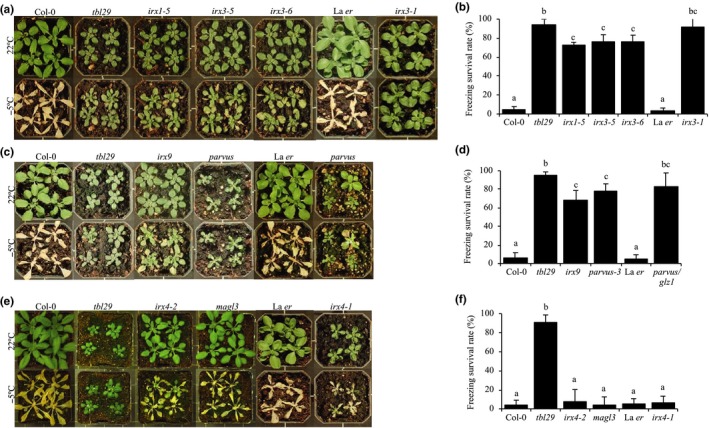
Freezing tolerance in *irregular xylem* mutants. (a, c, and e) Representative pictures of 4‐week‐old plants of the indicated genotypes before (upper panel) and after (lower panel) freezing treatment. (b, d, and f) Survival rate after freezing assay. Data are represented as mean (AVG) ± the standard deviation (*SD*) of three independent experiments (≥20 plants/experiment). Means with different letters are significantly different (Tukey's HSD,* p* < 0.05) in the significance (S) column

Similar to reduced xylan *O*‐acetylation in the *tbl29* Arabidopsis mutant, other secondary cell wall defects impact xylem morphology, plant size, and architecture. Because the *tbl29* mutant exhibits freezing tolerance, a potential general correlation of the *irregular xylem* phenotype with increased freezing tolerance was investigated (Figure 1). For this purpose, Arabidopsis T‐DNA insertion lines were obtained for genes involved in the synthesis of cellulose, xylan, and lignin. The *irregular xylem 1* (*irx1*) and *irregular xylem 3* (*irx3*) mutants have a severe deficiency in the deposition of cellulose in secondary walls, caused by mutations in the *CELLULOSE SYNTHASE 8* and *7* genes, respectively (Taylor, Laurie, & Turner, 2000). The *irregular xylem 9* (*irx9*) and *parvus* mutants are affected in the synthesis of the β‐1‐4‐xylan backbone and the tetrasaccharide structure located at the reducing end of xylan, respectively (Bauer, Vasu, Persson, Mort, & Somerville, 2006; Brown et al., 2007; Lee, O'Neill, Tsumuraya, Darvill, & Ye, 2007; Lee, Zhong, et al., 2007; Peña et al., 2007). Mutant alleles of *CINNAMOYL COA REDUCTASE 1* (*IRX4/CCR1*) and *CAFFEOYL SHIKIMATE ESTERASE* (*MAGL3/CSE/LYSOPL2*) genes affect lignin deposition (Jones et al., 2001; Vanholme et al., 2013). All of these mutants share with *tbl29* various developmental defects including reduced rosette size, a dwarfed stature, and a characteristic dark green color in the leaves (Figure 1). When freezing tolerance was evaluated, all tested alleles of cellulose‐ (*irx1* and *irx3,* Figure 1a,b) and xylan‐compromised mutants (*irx9* and *parvus;* Figure 1c,d) showed an increase in freezing tolerance compared to their respective controls. Although the *tbl29* mutants showed the highest survival rate (>90%) under the freezing conditions used, *irx1*,* irx3*,* irx9,* and *parvus* mutant lines also showed high values (>70%) compared to the respective controls (<10% survival rate). Interestingly, the two lignin‐compromised mutants analyzed (i.e., *irx4* and *magl3/cse;* Figure 1e,f) showed a wildtype‐like response in our freezing assay despite dwarfism and collapsed vessel elements (Jones et al., 2001; Vanholme et al., 2013). These results could be reproduced in a second Arabidopsis accession (Landsberg (La*er*)), at least for *irx3*,* irx4*, and *parvus*, where mutant alleles were available.

### 3.2 Effect of *max4* on other secondary wall mutants

**Figure 2:**
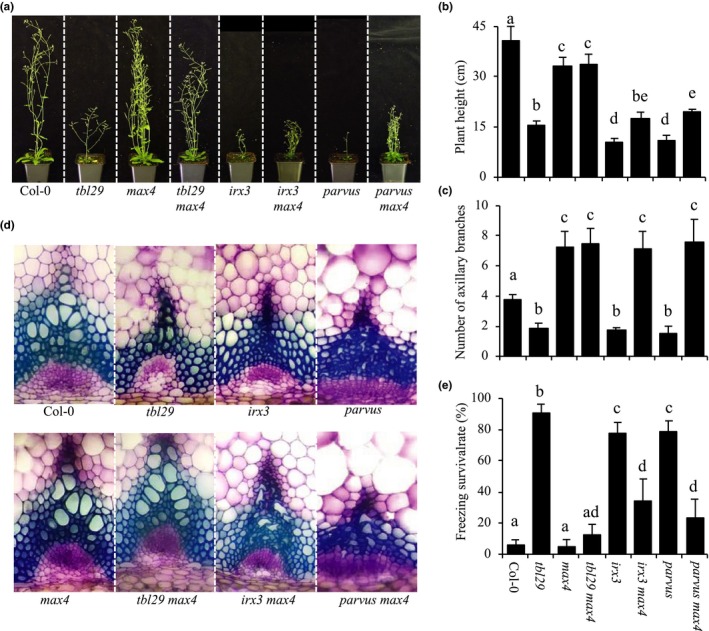
*max4* mutation in other secondary cell wall mutants. (a) Growth phenotypes of 6‐week‐old plants. (b) Primary inflorescence heights (cm). (c) Number of rosette branches. (d) Toluidine‐O‐Blue stained cross section of inflorescence stems. (e) Survival rate of freezing assay. Means with different letters are significantly different (Tukey's HSD,* p* < 0.05)

**Figure 3:**
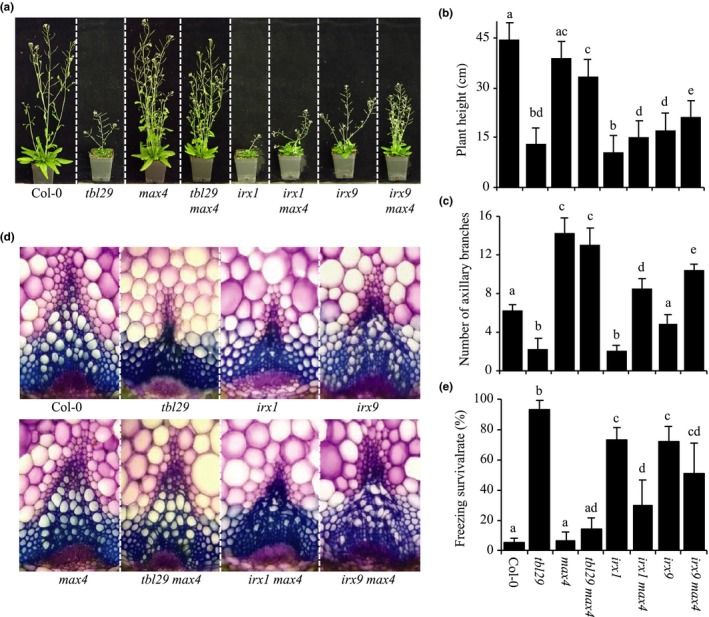
*max4* mutation in other secondary cell wall mutants. (a) Growth phenotypes of 6‐week‐old plants. (b) Primary inflorescence heights (cm). (c) Number of rosette branches. (d) Toluidine‐O‐Blue stained cross section of inflorescence stems. (e) Survival rate of freezing assay. Means with different letters are significantly different (Tukey's HSD,* p* < 0.05)

Mutations in the *MAX4* SL‐biosynthetic gene rescue the dwarfism, collapsed xylem, and increased freezing tolerance phenotypes associated with defects in xylan *O*‐acetylation in *tbl29* (Ramírez et al., 2018). The effect of the *max4* mutation on the phenotype of freezing tolerant cellulose‐ (i.e., *irx1* and *irx3*) and xylan‐ (i.e., *irx9* and *parvus*) deficient mutants was analyzed (Figures 2 and 3). Detailed analyses of the growth habit of the respective double mutants generated showed that the presence of *max4* leads to an increase in plant size in *irx1 max4*,* irx3 max4*,* irx9 max4*, and *parvus max4* plants. However, these double mutants do not exhibit a fully restored wildtype growth as was the case in *tbl29 max4* plants (Figures 2a,b and 3a,b). The increase in plant size is also accompanied by an increase in the number of rosette branches in the mutants (Figures 2c and 3c). A modest improvement is also observed in the collapsed xylem morphology in stem sections (Figures 2d and 3d). Although most of the xylem still remains irregular, more larger vessels are observed in the double mutants compared to the single mutants.

Similarly, the freezing tolerance in *irx1 max4*,* irx3 max4*, and *parvus max4* is significantly reduced compared to *irx1*,* irx3*, and *parvus* although the recovery is not complete as indicated by the intermediate survival rates of the double mutants (Figures 2e and 3e). In the case of *irx9* and *irx9 max4*, no significant reduction in freezing tolerance was observed.

Wall analyses showed no obvious differences in the composition of matrix polysaccharides caused by the introduction of *max4* into the various mutant backgrounds (Tables S1 and S2), and the defects caused by the *irx* mutations remain intact in the respective double mutants. However, a minor increase in cellulose content was noticed in *tbl29 max4*,* irx1 max4*,* irx3 max4*,* irx9 max4*, and *parvus max4* compared to the respective *tbl29*,* irx1*,* irx3, irx9*, and *parvus* single mutants.

Taken together, these results indicate that although *max4* is able to override the consequences of xylan hypoacetylation on plant development and freezing tolerance, it is only able to partially compensate for the more severe wall defects in the xylem vessels of reduced cellulose and altered xylan mutants.

### 3.3 Effect of *max4* on primary wall mutants

**Figure 4:**
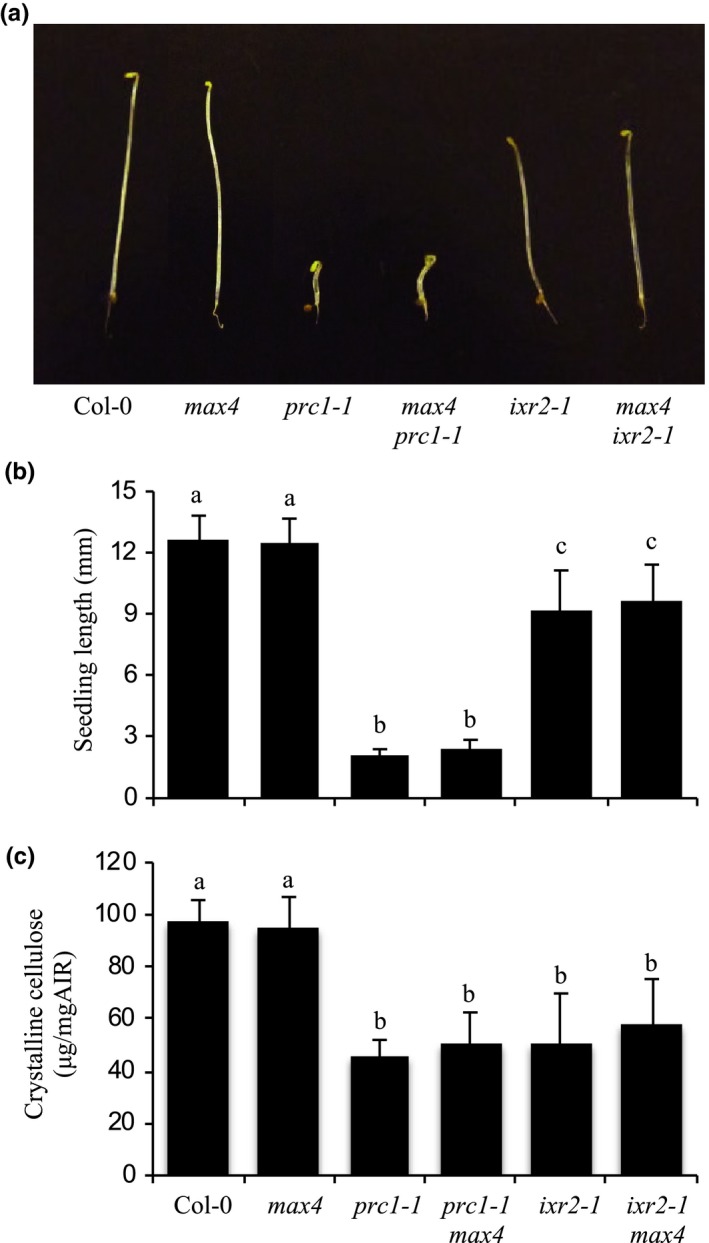
*max4* mutation in primary wall‐deficient mutants. (a) Representative pictures of dark‐grown 5‐day‐old seedlings. (b) Seedling length. Bars represent mean (AVG) ± the standard deviation (*SD*) of individual seedlings (*n* ≥ 100). (c) Crystalline cellulose content. Bars represent mean (AVG) ± the standard deviation (*SD*) of *n* = 5 biological replicates. Means with different letters are significantly different (Tukey's HSD,* p* < 0.05)

Defects in primary wall cellulose biosynthesis also lead to developmental defects including dwarfism of etiolated hypocotyls (Fagard et al., 2000; Scheible, Eshed, Richmond, Delmer, & Somerville, 2001). In order to evaluate the effect of blocking SL synthesis on primary wall‐deficient mutants, the *max4* mutation was introduced in two mutant alleles of the *PROCUSTE 1/ISOXABEN RESISTANT 2/CELLULOSE SYNTHASE 6* gene (*PRC1/IXR2/CesA6*). The *prc1‐1* strong allele shows severe reductions in cellulose deposition in the primary wall. As a result, seedling length is reduced by more than 80% compared to the wildtype. A weaker allele, *ixr2‐1,* only shows a mild reduction of seedling growth (≈25%). Introduction of *max4* mutation in *prc1‐1* or *ixr2‐1* mutant backgrounds does not complement the developmental defects, as the seedling length of double mutant plants remains reduced (Figure 4a,b). As expected, the presence of *max4* does not change the crystalline cellulose content on any of the two mutant backgrounds (Figure 4c). These results suggest that the dwarfism triggered by primary wall deficiencies is SL‐independent.

### 3.4 Effect of SL biosynthesis‐ and perception‐deficient mutants on *tbl29*‐associated *irregular xylem* phenotype

**Figure 5:**
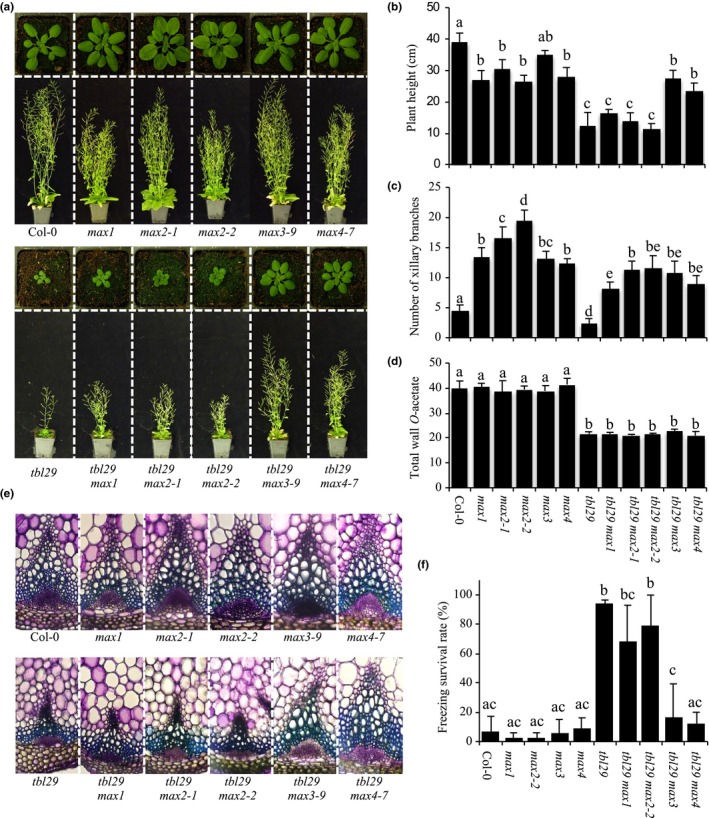
*max* mutations in tbl29. (a) Growth phenotypes of 4‐ (upper panel) and 6‐week‐old (lower panel) plants. (b) Primary inflorescence heights (cm). (c) Number of rosette branches. (d) Total wall O‐acetate in plant stems. (e) Toluidine‐O‐Blue stained cross section of inflorescence stems. (f) Survival rate of freezing assay. Means with different letters are significantly different (Tukey's HSD,* p* < 0.05)

The effect of blocking different steps of the SL pathway was investigated by introducing mutant alleles of *MAX1*,* MAX2*, and *MAX3* genes into the *tbl29* mutant background to generate the corresponding *tbl29 max1*,* tbl29 max2*, and *tbl29 max3* double mutants. A detailed characterization of these plants including the previously characterized *tbl29 max4* double mutant line was performed in terms of plant growth habit, xylem morphology, and freezing tolerance (Figure 5). Plant height in *tbl29 max3* and *tbl29 max4* double mutant was comparable to the *max3* and *max4* single mutants, rescuing almost completely the *tbl29* dwarf phenotype. On the other hand, *tbl29 max1* and *tbl29 max2* double mutant plants were still dwarfed, with stem heights similar to *tbl29* plants. This result was confirmed with two different *max2* mutant alleles (i.e., *max2‐1* and *max2‐2*).

Toluidine blue staining of stem sections of *max1*,* max2*,* max3*, and *max4* single mutants showed no obvious differences with Col‐0. While *tbl29 max3* and *tbl29 max4* double mutant plants also showed normal xylem development, equivalent stem sections of *tbl29 max1*,* tbl29 max2‐1*, and *tbl29 max2‐2* double mutant plants showed a collapsed xylem comparable to *tbl29* single mutant (Figure 5e). As previously shown for *tbl29 max4*, the rescued developmental defects in *tbl29 max3* are not associated with the recovery of normal xylan *O*‐acetylation levels as both double mutants show reduced values comparable to *tbl29* single mutant (Figure 5d).

No significant differences were found in the response to freezing temperatures of *max1*,* max2*,* max3*, and *max4* single mutants compared to Col‐0, with survival rates below 15%. In contrast, *tbl29 max1* and *tbl29 max2* plants were highly tolerant to freezing temperatures, with a survival rate over 70% similar to *tbl29*. As with plant height, the increased freezing tolerance associated with *tbl29* was suppressed in *tbl29 max3* and *tbl29 max4* double mutants with a survival rate comparable to Col‐0 (Figure 5f).

As previously reported, *tbl29* mutant plants exhibited a reduced number of axillary branches compared to Col‐0. On the other hand, *max* mutants showed an increased branching phenotype due to SL deficiency (Sorefan et al., 2003). All *tbl29 max* double mutants generated showed significantly increased number of axillary branches compared to *tbl29*, regardless of whether the plant height was rescued or not (Figure 5c). This result strongly suggests that the suppression of *tbl29* phenotypes observed in *tbl29 max3* and *tbl29 max4* is not due to an indirect effect of a change in plant architecture (i.e., increased number of axillary branches). Together, these results indicate that blocking any of the two CCDs (i.e., MAX3 and MAX4) involved in carlactone biosynthesis rescues the collapsed xylem, growth defects, and increased freezing tolerance associated with the reduction of xylan *O*‐acetylation caused by *TBL29* loss of function. On the other hand, mutations in genes encoding downstream enzymes in the SL pathway failed to complement the *tbl29* mutant phenotypes, suggesting that production (i.e., MAX1) or perception (i.e., MAX2) of other SL compounds is not required.

## 4 DISCUSSION

### 4.1 Freezing tolerance in secondary wall‐deficient mutants

A side‐by‐side analysis of multiple secondary wall‐deficient mutants showed that increased freezing tolerance seems to be a common phenotype of plants with altered xylan composition and cellulose content (Figure 1). The increased freezing tolerance of *tbl29/esk1* mutant alleles has been known for 20 years (Xin & Browse, 1998). Since then, various *tbl29* stress‐related phenotypes have been the focus of several studies (Bouchabke‐Coussa et al., 2008; Lefebvre et al., 2011; Xin et al., 2007). Similarly, various reports have also shown stress‐related phenotypes for individual secondary wall‐deficient mutants such as increased tolerance to salt stress, drought, or pathogen attack (Chen et al., 2005; Hernández‐Blanco et al., 2007; Keppler & Showalter, 2010; Ramírez et al., 2011). Although the mechanism by which *tbl29*/*esk1* plants become freezing tolerant is unknown, evidence has been presented that it is independent from the classical ABA hormonal pathway or the ICE1–CBF cold response pathway (Lefebvre et al., 2011). The current hypothesis entails that the increased freezing tolerance (and other constitutive stress responses) in *tbl29/esk1* mutants is caused by a transpiration imbalance produced by the collapse of the xylem. The constitutive freezing tolerance phenotype of multiple secondary wall mutants suggests that the same mechanism could apply to xylan‐ and cellulose‐deficient mutants. Interestingly, the two lignin‐deficient mutants analyzed here exhibited a wildtype‐like response in our freezing test, suggesting that lignin defects leading to xylem collapse might not trigger the same mechanism.

### 4.2 Effect of *max4* in primary and secondary wall mutants

Recently, we showed how mutations in the *MAX4* SL‐biosynthetic gene where able to suppress the developmental‐ and stress‐related phenotypes caused by the reduction in the xylan *O*‐acetylation in *tbl29* (Ramírez et al., 2018). The severity of the *irx* syndrome in *tbl29* is similar to other xylan‐ and cellulose‐deficient mutants as *irx1*,* irx3*, or *parvus* in terms of dwarf stature, collapsed xylem, or increased freezing tolerance (Figures 2 and 3). Introducing the *max4* mutation in these secondary wall mutants has a positive effect in plant height and collapsed xylem and partially rescues the constitutive freezing tolerance compared to the single mutants (Figures 2 and 3). These data suggest that, in general, secondary wall polysaccharide defects might trigger the SL pathway without repairing the defective wall structures or other structural compensations. However, although knocking out MAX4 recovers the *irx* syndrome caused by a reduction in xylan *O*‐acetylation, it is not able to restore completely the more severe secondary wall defects such as reduced cellulose content or defects in xylan backbone biosynthesis. It thus seems that in the case of minor structural modifications (i.e., reduced xylan *O*‐acetylation), *max4* mutation is able to short circuit the wall defect‐triggered signal and rescue the *irregular xylem*‐derived phenotypes. In contrast, when the defect compromises the secondary wall integrity/functionality (i.e., defects in the xylan backbone or cellulose synthesis), the rescue is only partial.

Multiple lines of evidence indicate the existence of a mechanism monitoring the integrity of primary walls (reviewed by Wolf, 2017). Primary wall‐deficient mutants display retarded growth due to defective cellulose deposition and can be complemented by blocking components of a still not completely understood signaling network without affecting the wall composition (Fagard et al., 2000; Scheible et al., 2001; Wolf, 2017). Similar to the case of *tbl29*, it seems that the developmental phenotypes exhibited by these primary wall mutants (e.g., *prc1* or *ixr2*) are not a direct consequence of the wall defect but the activation of a downstream signaling response. Introduction of *max4* mutation in strong and mild mutant alleles of a primary wall‐specific cellulose synthase (i.e., *CesA6)* does not complement the developmental defects (Figure 4), suggesting that in the primary cell wall integrity system, the SL pathway is not involved, and MAX4 has a secondary wall‐specific function likely restricted to vascular tissue.

### 4.3 Specificity of *max* mutations as suppressors of *tbl29*

**Figure 6:**
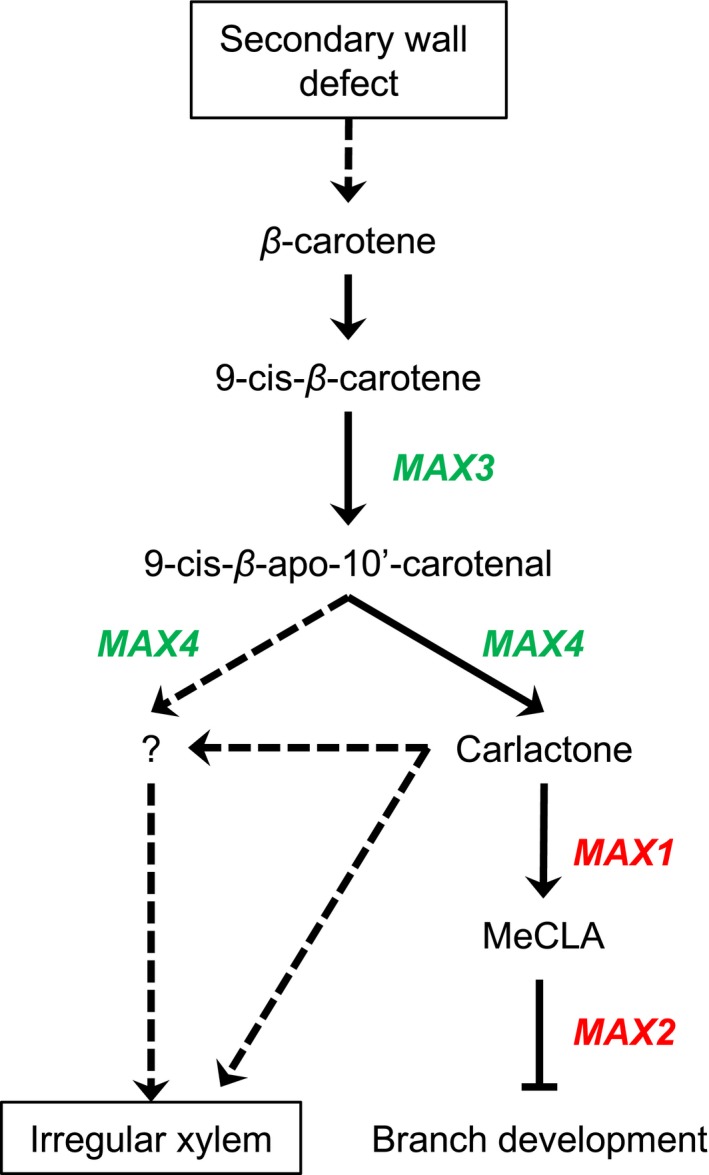
A genetic model of the interaction between secondary wall defects and the SL biosynthetic pathway. SL biosynthesis begins with the conversion of β‐carotene into carlactone by the consecutive action of MAX3 and MAX4. Carlactone then acts as a precursor for the synthesis of species‐specific SL compounds. In Arabidopsis, biosynthesis and perception of MeCLA, a major active SL, have been well characterized. Carlactone is converted to carlactonoic acid by MAX1 and then to MeCLA by an unknown enzyme. MeCLA is then perceived by a receptor complex including MAX2 in order to regulate several plant processes (e.g., branch development). According to our model, secondary wall defects caused by alterations in cellulose and xylan biosynthesis trigger a *irregular xylem* syndrome as observed in the *tbl29* mutant. Mutations in the MAX3 and MAX4 genes (in green) are able to suppress *tbl29* suggesting that production of carlactone is required for the xylem collapse in this wall‐deficient mutant. In contrast, mutations in *MAX1* or *MAX2* (in red) fail to complement *tbl29*, suggesting that downstream steps in the MAX1‐mediated biosynthesis and MAX2‐mediated perception of SL would not be involved in this signaling. Dashed arrows indicate unknown steps. First, it is still unknown how secondary wall defects trigger the activation of the SL pathway. Second, it is unclear if carlactone directly or an unknown carlactone‐derived molecule regulates xylem development. Finally, we cannot exclude the possibility that MAX4 catalyzes the synthesis of a related compound/s (?), not necessarily derived from carlactone, regulating xylem development

While *max3* and *max4* mutations were able to suppress dwarfism, collapsed xylem, and increased freezing tolerance associated with *tbl29*, other mutations causing SL deficiency (*max1*) or SL insensitivity (*max2*) failed to complement (Figure 5). Considering that all *max* mutations induced an increased axillary shoot branching in the *tbl29* mutant background regardless of the suppression of the *tbl29*‐associated phenotypes, we can conclude that this phenomenon is not due to an altered plant architecture induced by SL deficiency. Our genetic data, summarized in Figure 6, suggest that specifically some components of the SL‐biosynthetic pathway are involved. MAX3 and MAX4 encode two CCD enzymes, whose sequential actions produce carlactone from β‐carotene (Alder et al., 2012; Auldridge et al., 2006; Matusova et al., 2005). According to our results, endogenous production of carlactone would be required for the expression of developmental‐ and stress‐related phenotypes caused by certain secondary wall defects (e.g., *tbl29*). Intriguingly, it seems that this effect is independent on downstream steps in the SL biosynthesis such as MAX1 and MAX2. These results apparently contradict the current SL model of action, based on the SL‐dependent inhibition of shoot branching. Our genetic data imply that carlactone has a regulatory role independent on the perception of SL compounds through MAX2. However, this is not the first result contradicting this model. For example, carlactone applications are not only able to complement the lateral inflorescence phenotype in SL‐deficient mutants (e.g., *max1* and *max4*) but also partially in *max2* SL‐insensitive mutant plants, suggesting the existence of a MAX2‐independent perception mechanism for carlactone. In contrast, MeCLA and CLA applications complement only SL‐deficient mutants but not in *max2*, indicating that unlike carlactone, MeCLA/CLA perception requires MAX2 (Abe et al., 2014). Carlactone has been proposed as a precursor of other SL compounds, and subsequent species‐specific rearrangements and modifications catalyzed by downstream enzymes would convert carlactone into the large diversity of SL compounds found in plants (Iseki et al., 2018; Seto et al., 2014). Thus, we cannot rule out the possibility that not carlactone itself, but a carlactone‐derived, MAX1‐independent SL compound is involved in this regulation of xylem development in secondary wall‐deficient mutants. Another possibility could be that in addition to carlactone, MAX3 and MAX4 catalyze the biosynthesis of hitherto unidentified molecules related to SL that regulate the *irregular xylem* syndrome triggered by secondary wall deficiencies. In this regard, it has been proposed that MAX3 and MAX4 could be involved in the enzymatic and non‐enzymatic cleavage of carotenoid substrates other than β‐carotene to produce biologically important derivatives (reviewed in Hou, Rivers, Leon, McQuinn, & Pogson, 2016). Future experiments evaluating the requirement of other components involved in carlactone biosynthesis (e.g., AtD27) and specific SL perception (e.g., D14) are needed in order to distinguish between these possibilities.

## CONFLICT OF INTEREST

The authors declare no conflict of interest associated with the work described in this manuscript.

## AUTHOR CONTRIBUTIONS

M.P. and V.R. designed the research; V.R. conducted experiments; M.P. and V.R. wrote the paper.

## References

[pld3149-bib-0001] Abe, S. , Sado, A. , Tanaka, K. , Kisugi, T. , Asami, K. , Ota, S. , … Nomura, T. (2014). Carlactone is converted to carlactonoic acid by MAX1 in Arabidopsis and its methyl ester can directly interact with AtD14 in vitro. Proceedings of the National Academy of Sciences of the United States of America, 111, 18084–18089. 25425668 10.1073/pnas.1410801111 PMC4273391

[pld3149-bib-0002] Al‐Babili, S. , & Bouwmeester, H. J. (2015). Strigolactones, a novel carotenoid‐derived plant hormone. Annual Review of Plant Biology, 66, 161–186. 10.1146/annurev-arplant-043014-114759 25621512

[pld3149-bib-0003] Alder, A. , Jamil, M. , Marzorati, M. , Bruno, M. , Vermathen, M. , Bigler, P. , … Al‐Babili, S. (2012). The path from beta‐carotene to carlactone, a strigolactone‐like plant hormone. Science, 335, 1348–1351. 22422982 10.1126/science.1218094

[pld3149-bib-0004] Arite, T. , Iwata, H. , Ohshima, K. , Maekawa, M. , Nakajima, M. , Kojima, M. , … Kyozuka, J. (2007). DWARF10, an RMS1/MAX4/DAD1 ortholog, controls lateral bud outgrowth in rice. Plant Journal, 51, 1019–1029. 17655651 10.1111/j.1365-313X.2007.03210.x

[pld3149-bib-0005] Auldridge, M. E. , Block, A. , Vogel, J. T. , Dabney‐Smith, C. , Mila, I. , Bouzayen, M. , … Klee, H. J. (2006). Characterisation of three members of the Arabidopsis carotenoid cleavage dioxygenase family demonstrates the divergent roles of this multifunctional enzyme family. Plant Journal, 45, 982–993. 16507088 10.1111/j.1365-313X.2006.02666.x

[pld3149-bib-0006] Bauer, S. , Vasu, P. , Persson, S. , Mort, A. J. , & Somerville, C. R. (2006). Development and application of a suite of polysaccharide‐degrading enzymes for analyzing plant cell walls. Proceedings of the National Academy of Sciences of the United States of America, 10, 11417–11422. 10.1073/pnas.0604632103 PMC1544100 16844780

[pld3149-bib-0007] Bennett, T. , Liang, Y. , Seale, M. , Ward, S. , Müller, D. , & Leyser, O. (2016). Strigolactone regulates shoot development through a core signalling pathway. Biology Open, 15, 1806–1820. 10.1242/bio.021402 PMC5200909 27793831

[pld3149-bib-0008] Bensussan, M. , Lefebvre, V. , Ducamp, A. , Trouverie, J. , Gineau, E. , Fortabat, M. N. , … Durand‐Tardif, M. (2015). Suppression of dwarf and irregular xylem phenotypes generates low‐acetylated biomass lines in Arabidopsis. Plant Physiology, 168, 452–463. 25888614 10.1104/pp.15.00122 PMC4453781

[pld3149-bib-0009] Booker, J. , Auldridge, M. , Wills, S. , McCarty, D. , Klee, H. , & Leyser, O. (2004). MAX3/CCD7 is a carotenoid cleavage dioxygenase required for the synthesis of a novel plant signaling molecule. Current Biology, 14, 1232–1238. 15268852 10.1016/j.cub.2004.06.061

[pld3149-bib-0010] Bouchabke‐Coussa, O. , Quashie, M. L. , Seoane‐Redondo, J. , Fortabat, M. N. , Gery, C. , Yu, A. , … Durand‐Tardif, M. (2008). ESKIMO1 is a key gene involved in water economy as well as cold acclimation and salt tolerance. BMC Plant Biology, 8, 125–148. 19061521 10.1186/1471-2229-8-125 PMC2630945

[pld3149-bib-0011] Brown, D. M. , Goubet, F. , Wong, V. W. , Goodacre, R. , Stephens, E. , Dupree, P. , & Turner, S. R. (2007). Comparison of five xylan synthesis mutants reveals new insight into the mechanisms of xylan synthesis. Plant Journal, 52, 1154–1168. 17944810 10.1111/j.1365-313X.2007.03307.x

[pld3149-bib-0012] Brown, D. M. , Zeef, L. A. H. , Ellis, J. , Goodacre, R. , & Turner, S. R. (2005). Identification of novel genes in Arabidopsis involved in secondary cell wall formation using expression profiling and reverse genetics. Plant Cell, 17, 2281–2295. 15980264 10.1105/tpc.105.031542 PMC1182489

[pld3149-bib-0013] Chen, Z. , Hong, X. , Zhang, H. , Wang, Y. , Li, X. , Zhu, J. K. , & Gong, Z. (2005). Disruption of the cellulose synthase gene, AtCesA8/IRX1, enhances drought and osmotic stress tolerance in Arabidopsis. Plant Journal, 43, 273–283. 15998313 10.1111/j.1365-313X.2005.02452.x

[pld3149-bib-0014] Downes, B. P. , Stupar, R. M. , Gingerich, D. J. , & Vierstra, R. D. (2003). The HECT ubiquitin‐protein ligase (UPL) family in Arabidopsis: UPL3 has a specific role in trichome development. Plant Journal, 35, 729–742. 12969426 10.1046/j.1365-313x.2003.01844.x

[pld3149-bib-0015] El Refy, A. , Perazza, D. , Zekraoui, L. , Valay, J. G. , Bechtold, N. , Brown, S. , … Bonneville, J. M. (2003). The Arabidopsis KAKTUS gene encodes a HECT protein and controls the number of endoreduplication cycles. Molecular Genetics and Genomics, 270, 403–414. 14530964 10.1007/s00438-003-0932-1

[pld3149-bib-0016] Fagard, M. , Desnos, T. , Desprez, T. , Goubet, F. , Refregier, G. , Mouille, G. , … Höfte, H. (2000). PROCUSTE1 encodes a cellulose synthase required for normal cell elongation specifically in roots and dark‐grown hypocotyls of Arabidopsis. Plant Cell, 12, 2409–2424. 11148287 10.1105/tpc.12.12.2409 PMC102227

[pld3149-bib-0017] Foster, C. E. , Martin, T. M. , & Pauly, M. (2010). Comprehensive compositional analysis of plant cell walls (lignocellulosic biomass) Part II: Carbohydrates. Journal of Visualized Experiments, 37, e1745. 10.3791/1837 PMC3145335 20228730

[pld3149-bib-0018] Hernández‐Blanco, C. , Feng, D. X. , Hu, J. , Sánchez‐Vallet, A. , Deslandes, L. , Llorente, F. , … Molina, A. (2007). Impairment of cellulose synthases required for Arabidopsis secondary cell wall formation enhances disease resistance. Plant Cell, 19, 890–903. 17351116 10.1105/tpc.106.048058 PMC1867366

[pld3149-bib-0019] Hou, X. , Rivers, J. , Leon, P. , McQuinn, R. P. , & Pogson, B. J. (2016). Synthesis and function of apocarotenoid signals in plants. Trends in Plant Science, 21, 792–803. 27344539 10.1016/j.tplants.2016.06.001

[pld3149-bib-0020] Iseki, M. , Shida, K. , Kuwabara, K. , Wakabayashi, T. , Mizutani, M. , Takikawa, H. , & Sugimoto, Y. (2018). Evidence for species‐dependent biosynthetic pathways for converting carlactone to strigolactones in plants. Journal of Experimental Botany, 69, 2305–2318. 29294064 10.1093/jxb/erx428 PMC5913628

[pld3149-bib-0021] Jiang, L. , Liu, X. , Xiong, G. , Liu, H. , Chen, F. , Wang, L. , … Li, J. (2013). DWARF 53 acts as a repressor of strigolactone signalling in rice. Nature, 504, 401–405. 24336200 10.1038/nature12870 PMC5802366

[pld3149-bib-0022] Johnson, X. , Brcich, T. , Dun, E. A. , Goussot, M. , Haurogné, K. , Beveridge, C. A. , & Rameau, C. (2006). Branching genes are conserved across species. Genes controlling a novel signal in pea are coregulated by other long‐distance signals. Plant Physiology, 142, 1014–1026. 16980559 10.1104/pp.106.087676 PMC1630745

[pld3149-bib-0023] Jones, L. , Ennos, A. R. , & Turner, S. R. (2001). Cloning and characterization of irregular xylem4 (irx4): A severely lignin‐deficient mutant of Arabidopsis. Plant Journal, 26, 205–216. 11389761 10.1046/j.1365-313x.2001.01021.x

[pld3149-bib-0024] Keppler, B. D. , & Showalter, A. M. (2010). IRX14 and IRX14‐LIKE, two glycosyl transferases involved in glucuronoxylan biosynthesis and drought tolerance in Arabidopsis. Molecular Plant, 3(5), 834–841. 20595206 10.1093/mp/ssq028

[pld3149-bib-0025] Lee, C. H. , O'Neill, M. A. , Tsumuraya, Y. , Darvill, A. G. , & Ye, Z. H. (2007). The irregular xylem9 mutant is deficient in xylan xylosyltransferase activity. Plant and Cell Physiology, 48, 1624–1634. 17938130 10.1093/pcp/pcm135

[pld3149-bib-0026] Lee, C. H. , Zhong, R. Q. , Richardson, E. A. , Himmelsbach, D. S. , McPhail, B. T. , & Ye, Z. H. (2007). The PARVUS gene is expressed in cells undergoing secondary wall thickening and is essential for glucuronoxylan biosynthesis. Plant and Cell Physiology, 48, 1659–1672. 17991630 10.1093/pcp/pcm155

[pld3149-bib-0027] Lefebvre, V. , Fortabat, M. N. , Ducamp, A. , North, H. M. , Maia‐Grondard, A. , Trouverie, J. , … Durand‐Tardif, M. (2011). ESKIMO1 disruption in Arabidopsis alters vascular tissue and impairs water transport. PLoS ONE, 6, e16645. 21408051 10.1371/journal.pone.0016645 PMC3052256

[pld3149-bib-0028] Matusova, R. , Rani, K. , Verstappen, F. W. A. , Franssen, M. C. R. , Beale, M. H. , & Bouwmeester, H. J. (2005). The strigolactone germination stimulants of the plant‐parasitic Striga and Orobanche spp. are derived from the carotenoid pathway. Plant Physiology, 139, 920–934. 16183851 10.1104/pp.105.061382 PMC1256006

[pld3149-bib-0029] Peña, M. J. , Zhong, R. Q. , Zhou, G. K. , Richardson, E. A. , O'Neill, M. A. , Darvill, A. G. , … Ye, Z. H. (2007). Arabidopsis irregular xylem8 and irregular xylem9: Implications for the complexity of glucuronoxylan biosynthesis. Plant Cell, 19, 549–563. 17322407 10.1105/tpc.106.049320 PMC1867335

[pld3149-bib-0030] Ramírez, V. , Agorio, A. , Coego, A. , García‐Andrade, J. , Hernández, M. J. , Balaguer, B. , … Vera, P. (2011). MYB46 modulates disease susceptibility to Botrytis cinerea in Arabidopsis. Plant Physiology, 155, 1–16. 21282403 10.1104/pp.110.171843 PMC3091096

[pld3149-bib-0031] Ramírez, V. , Xiong, G. , Mashiguchi, K. , Yamaguchi, S. , & Pauly, M. (2018). Growth‐ and stress‐related defects associated with wall hypoacetylation are strigolactone‐dependent. Plant Direct, 2, e00062. 10.1002/pld3.62 PMC6508513 31245725

[pld3149-bib-0032] Scaffidi, A. , Waters, M. T. , Ghisalberti, E. L. , Dixon, K. W. , Flematti, G. R. , & Smith, S. M. (2013). Carlactone‐independent seedling morphogenesis in Arabidopsis. Plant Journal, 76, 1–9. 23773129 10.1111/tpj.12265

[pld3149-bib-0033] Scheible, W. R. , Eshed, R. , Richmond, T. , Delmer, D. , & Somerville, C. (2001). Modifications of cellulose synthase confer resistance to isoxaben and thiazolidinone herbicides in Arabidopsis Ixr1 mutants. Proceedings of the National Academy of Sciences of the United States of America, 98, 10079–10084. 11517344 10.1073/pnas.191361598 PMC56918

[pld3149-bib-0034] Scholl, R. L. , May, S. T. , & Ware, D. H. (2000). Seed and molecular resources for Arabidopsis. Plant Physiology, 124(4), 1477–1480. 11115863 10.1104/pp.124.4.1477 PMC1539300

[pld3149-bib-0035] Schwartz, S. H. , Qin, X. , & Loewen, M. C. (2004). The biochemical characterization of two carotenoid cleavage enzymes from Arabidopsis indicates that a carotenoid‐derived compound inhibits lateral branching. Journal of Biological Chemistry, 279, 46940–46945. 15342640 10.1074/jbc.M409004200

[pld3149-bib-0036] Seto, Y. , Sado, A. , Asami, K. , Hanada, A. , Umehara, M. , Akiyama, K. , & Yamaguchi, S. (2014). Carlactone is an endogenous biosynthetic precursor for strigolactones. Proceedings of the National Academy of Sciences of the United States of America, 111, 1640–1645. 24434551 10.1073/pnas.1314805111 PMC3910621

[pld3149-bib-0037] Seto, Y. , Yasui, R. , Kameoka, H. , Tamiru, M. , Cao, M. , Terauchi, R. , … Yamaguchi, S. (2019). Strigolactone perception and deactivation by a hydrolase receptor DWARF14. Nature Communications, 10(1), 191. 10.1038/s41467-018-08124-7 PMC6331613 30643123

[pld3149-bib-0038] Snowden, K. C. , Simkin, A. J. , Janssen, B. J. , Templeton, K. R. , Loucas, H. M. , Simons, J. L. , … Klee, H. J. (2005). The Decreased apical dominance1/Petunia hybrida CAROTENOID CLEAVAGE DIOXYGENASE8 gene affects branch production and plays a role in leaf senescence, root growth, and flower development. Plant Cell, 17, 746–759. 15705953 10.1105/tpc.104.027714 PMC1069696

[pld3149-bib-0039] Sorefan, K. , Booker, J. , Haurogné, K. , Goussot, M. , Bainbridge, K. , Foo, E. , … Leyser, O. (2003). MAX4 and RMS1 are orthologous dioxygenase‐like genes that regulate shoot branching in Arabidopsis and pea. Genes & Development, 17, 1469–1474. 12815068 10.1101/gad.256603 PMC196077

[pld3149-bib-0040] Soundappan, I. , Bennett, T. , Morffy, N. , Liang, Y. , Stanga, J. P. , Abbas, A. , … Nelson, D. C. (2015). SMAX1‐LIKE/D53 family members enable distinct MAX2‐dependent responses to strigolactones and karrikins in Arabidopsis. Plant Cell, 27, 3143–3159. 26546447 10.1105/tpc.15.00562 PMC4682302

[pld3149-bib-0041] Taylor, N. G. , Laurie, S. , & Turner, S. R. (2000). Multiple cellulose synthase catalytic subunits are required for cellulose synthesis in Arabidopsis. Plant Cell, 12, 2529–2540. 11148295 10.1105/tpc.12.12.2529 PMC102235

[pld3149-bib-0042] Tokunaga, T. , Hayashi, H. , & Akiyama, K. (2015). Medicaol, a strigolactone identified as a putative didehydro‐orobanchol isomer, from *Medicago truncatula* . Phytochemistry, 111, 91–97. 25593009 10.1016/j.phytochem.2014.12.024

[pld3149-bib-0043] Turner, S. R. , & Somerville, C. R. (1997). Collapsed xylem phenotype of Arabidopsis identifies mutants deficient in cellulose deposition in the secondary cell wall. Plant Cell, 9, 689–701. 9165747 10.1105/tpc.9.5.689 PMC156949

[pld3149-bib-0044] Vanholme, R. , Cesarino, I. , Rataj, K. , Xiao, Y. , Sundin, L. , Goeminne, G. , … Boerjan, W. (2013). Caffeoyl shikimate esterase (CSE) is an enzyme in the lignin biosynthetic pathway in Arabidopsis. Science, 341(6150), 1103–1106. 23950498 10.1126/science.1241602

[pld3149-bib-0045] Wang, L. , Wang, B. , Jiang, L. , Liu, X. , Li, X. , Lu, Z. , … Li, J. (2015). Strigolactone signaling in Arabidopsis regulates shoot development by targeting D53‐like SMXL repressor proteins for ubiquitination and degradation. Plant Cell, 27, 3128–3142. 26546446 10.1105/tpc.15.00605 PMC4682305

[pld3149-bib-0046] Waters, M. T. , Gutjahr, C. , Bennett, T. , & Nelson, D. C. (2017). Strigolactone signaling and evolution. Annual Review of Plant Biology, 68, 291–322. 10.1146/annurev-arplant-042916-040925 28125281

[pld3149-bib-0047] Waters, M. T. , Nelson, D. C. , Scaffidi, A. , Flematti, G. R. , Sun, Y. K. , Dixon, K. W. , & Smith, S. M. (2012). Specialisation within the DWARF14 protein family confers distinct responses to karrikins and strigolactones in Arabidopsis. Development, 139, 1285–1295. 22357928 10.1242/dev.074567

[pld3149-bib-0048] Wolf, S. (2017). Plant cell wall signalling and receptor‐like kinases. Biochemical Journal, 15, 471–492. 10.1042/BCJ20160238 28159895

[pld3149-bib-0049] Xin, Z. , & Browse, J. (1998). Eskimo1 mutants of Arabidopsis are constitutively freezing‐tolerant. Proceedings of the National Academy of Sciences of the United States of America, 95, 7799–7804. 9636231 10.1073/pnas.95.13.7799 PMC22762

[pld3149-bib-0050] Xin, Z. , Mandaokar, A. , Chen, J. , Last, R. L. , & Browse, J. (2007). Arabidopsis ESK1 encodes a novel regulator of freezing tolerance. Plant Journal, 49, 786–799. 17316173 10.1111/j.1365-313X.2006.02994.x

[pld3149-bib-0051] Xiong, G. , Cheng, K. , & Pauly, M. (2013). Xylan O‐acetylation impacts xylem development and enzymatic recalcitrance as indicated by the Arabidopsis mutant tbl29. Molecular Plant, 6, 1373–1375. 23340742 10.1093/mp/sst014

[pld3149-bib-0052] Zou, J. H. , Zhang, S. Y. , Zhang, W. P. , Li, G. , Chen, Z. X. , Zhai, W. X. , … Zhu, L. H. (2006). The rice HIGH‐TILLERING DWARF1 encoding an ortholog of Arabidopsis MAX3 is required for negative regulation of the outgrowth of axillary buds. Plant Journal, 48, 687–698. 17092317 10.1111/j.1365-313X.2006.02916.x

