# Supplementary material for: Genetic dissection of cell wall defects and the strigolactone pathway in Arabidopsis
Source: Plant Direct. 2019 Jun 22;3(6):e00149. doi: 10.1002/pld3.149 (PMC6589044; doi:10.1002/pld3.149)
Supplement: Supplementary file 2 [file PLD3-3-e00149-s002.pdf]

Dear Editor,

Thank you for your and the reviewer's valuable comments. We are pleased to resubmit our improved manuscript entitled "Genetic dissection of cell wall defects and the strigolactone pathway in Arabidopsis" by Vicente Ramirez and Markus Pauly.

We have now prepared a revised version of the manuscript 2019-00271, where all the reviewer's comments and suggestions have been implemented (Please see below). As suggested by Reviewer#2, this new version introduces a new Figure 6 showing the steps of the SL biosynthesis and perception analyzed in our double mutant strategy. Moreover, we also introduced a model of the linked signaling pathway involved in the irregular xylem syndrome caused by secondary wall defects, including additional possibilities suggested by Reviewer#1.

We also revised our statements, re-phrasing the conclusion that a SL component has to be the effective molecule. In the revised version, we suggest the more general idea that a MAX4-dependent molecule is required, encompassing three possibilities: 1) the effective molecule may be carlactone, as the direct product of MAX4 action (when β-carotene is used as a substrate); 2) Not carlactone directly, but a carlactone-derived molecule; or 3) A MAX3/ MAX4-derived, carlactone-independent molecule (when unknown carotenoid/s are used as a substrate). We also included suggestions to the future experiments suggested by Reviewer#2 in order to distinguish between some of these different options.
We hope you find the revised version of the manuscript suitable for publication in Plant Direct, and look forward to hearing your response.

Sincerely,

Markus Pauly

Response to the reviewers

Reviewer #1:

In this article, the authors expand on their previous work, examining the unanticipated interaction between strigolactones and secondary cell wall components. The authors find that mutations in a range of secondary cell wall components confer freezing tolerance, but that this is revesed in genetic backgrounds with mutations in the strigolactone biosynthetic enzymes MAX3 and MAX4 (but not MAX1 or MAX2).

The work in the manuscript is a relatively straightforward combination of genetics and phenotyping, and the work seems to have been compently performed and well analysed. I have no real comments about the work involved. The manuscript is also well written and presented.

However, I am not convinced by the authors' main interpretation of their data. Indeed, I have to confess that I am somewhat confused by the authors' data per se. In the absence of any obvious problems in the experiments themselves, I accept that authors assertion that only max3 and max4 suppress the cell wall mutant phenotypes. But I am somewhat puzzled as to why this might be. In the absence of any better suggestion, I cannot disagree with the authors' conclusion that a MAX3/MAX4 co-product contributes to the xylem collapse in secondary cell wall mutants, in a MAX2-independent manner. Where I disagree with the authors is that this has anything to do with

strigolactone. Almost by definition, if it doesn't require MAX1 for synthesis or MAX2 for signalling, then it isn't a strigolactone at all. Every known effect of strigolactones is mediated by MAX2, so if this effect doesn't act through MAX2, it cannot be safely concluded that it is an effect of strigolactones.

Because of other similar phenotypic oddities, there have previously been quite a few suggestions in the field that there may be as-yet-unidentified molecules out there that are related to strigolactones (perhaps even sharing synthesis pathway components), but which are not strigolactones. It may be that the authors have inadvertantly stumbled across such a molecule through their work. NB that this would not necessarily be a carlactone-derived molecule. Synthesis of carlactone requires DWARF27 to convert all-trans B-carotene to 9-cis-B-carotene before MAX3 and MAX4 convert it to carlactone. However, MAX3 and MAX4 can also act on all-trans B-carotene, and the hypothetical novel molecule could be derived from that substrate instead.

In this respect it is a shame that the authors did not test whether d27 mutants can also suppress the phenotypes, as this would help distinguish between some of these possibilities. Similarly, it would have useful to include d14 mutants, since these are specific for strigolactone signalling, unlike max2.

Given the current data, the authors cannot safely conclude that it is indeed strigolactone that contributes to the strong phenotypes in secondary cell wall mutants. The manuscript therefore needs to be re-written to remove the suggestion throughout that this is an effect of strigolactones - either that, or the authors need to provide concrete data that this really is an effect of strigolactones.

We agree with Reviewer#2 in the sense that the results obtained are somehow puzzling. However, the phenotypes observed in the various double mutants generated are very clear. In the previous version of the manuscript we assumed that the main function/activity of MAX4 is the production of carlactone supported by extensive genetic and biochemical characterization in multiple reports. However, we accept the possibility that alternative functions/activities of MAX4 independent of carlatone synthesis might be involved. In the revised version of the manuscript, we now include an alternative explanation of the results suggested by Reviewer #1. We also introduced the future experiments suggested by Reviewer #1 as a possibility to distinguish between some of the different options presented. Accordingly, a new paragraph was introduced at the end of the discussion (top Pag. 7):
"Another possibility could be that, in addition to carlactone, MAX3 and MAX4 catalyze the biosynthesis of unidentified molecules related to SL that regulate the irregular xylem syndrome triggered by secondary wall deficiencies. In this regard, it has been proposed that MAX3 and MAX4 could be involved in the enzymatic and non-enzymatic cleavage of carotenoid substrates other than *β*-carotene to produce biologically important derivatives (Reviewed in Hou et al., 2016). Future experiments evaluating the requirement of other components involved in carlactone biosynthesis (e.g. AtD27) and specific SL perception (e.g. D14) are needed in order to distinguish between these possibilities.".
This alternative possibility was also introduced in the new Figure 6 (A genetic model of the interaction between secondary wall defects and the SL biosynthetic pathway) suggested by Reviewer #2.

We also re-wrote the text to remove the suggestion that only a strigolactone compound contributes to the strong phenotypes in secondary cell wall mutants. The text was modified to suggest a more general interpretation instead, where the responsible molecule is MAX4-dependent. We think this interpretation encompasses all discussed possibilities and is more accurately supported by the data presented in the manuscript. Please notice the following modifications:

- In the Abstract: "the *irx* syndrome in the *trichome birefringence-like 29/eskimo1* (*tbl29/esk1*) mutant is dependent on the biosynthesis of the phytohormone strigolactone (SL)" was changed to "the *irx* syndrome in the *trichome birefringence-like 29/eskimo1* (*tbl29/esk1*) mutant is dependent on MORE AXILLARY GROWTH 4 (MAX4)"
- Also in the abstract: "blocking SL synthesis has also a suppressor effect on these phenotypes" was changed to "…these phenotypes are also dependent on MAX4…"
- Last paragraph in the abstract: "Our results are consistent with a specific role for carlactone in this process, and suggest that a MORE AXILLARY GROWTH 2 (MAX2)-independent SL perception mechanism might be involved." Was changed to "Our results show that the *tbl29*-associated *irx* phenotypes are dependent on the MAX3 and MAX4 enzymes, involved in the early steps of SL biosynthesis. In contrast, this signaling is independent on downstream enzymes in the biosynthesis and perception of SL such as MAX1 and MAX2."
- Results and discussion subsection titles were re-phrased changing "Effect of SL-deficiency …" for a more general "Effect of *max4* …"
- In the discussion (Pag. 6), the following sentences were re-written:
"...blocking SL biosynthesis is able to short circuit the wall defect-triggered signal and rescue the irregular xylem-derived phenotypes." was changed to "...*max4* mutation is able to short circuit the wall defect-triggered signal and rescue the irregular xylem-derived phenotypes."
"although blocking SL biosynthesis recovers the *irx* syndrome caused by a reduction in xylan *O*-acetylation..." was changed to "...although knocking out MAX4 recovers the *irx* syndrome caused by a reduction in xylan *O*-acetylation…"
"…but the SL pathway has a secondary wall-specific function likely restricted to vascular tissue" was changed to "…and MAX4 has a secondary wall-specific function likely restricted to vascular tissue"

Minor points:
1) Top of page 3: It has been demonstrated that DELLA proteins and BES1 are not targets of SL signalling at all, and that all effects of SL can be explained by degradation of SMXL6/7/8. See Bennett et al, 2016 (Biology Open).

The sentence describing DELLA proteins and BES1 as targets of SL signaling was removed from the text, together with the corresponding references Nakamura at al., 2013 Nat. Commun. and Wang et al., 2013, Dev. Cell. A new reference was introduced (Bennett et al., 2016. Biol. Open) as suggested.

Reviewer #2:

Overview
Plant cell walls are complex polysaccharide-rich extracellular matrices that surround all plant cells. Additionally, certain types of cells, such as xylem in the vascular tissue, produce secondary cell wall thickenings. Secondary cell walls consist primarily of cellulose, hemicelluloses, and lignin, which are used in aggregate to provide substantial mechanical support to the secondary cell wall. Several Arabidopsis mutants have been isolated that are impaired in cellulose, hemicellulose, or lignin biosynthesis specifically in secondary cell walls, and each of these mutants exhibit characteristic phenotypes, including shorter plant stature and irregularly shaped xylem cells, suggesting that defects in secondary cell wall biosynthesis broadly compromise these processes.
In this study, the authors build upon a previous finding that mutations in the MAX4 gene, which is implicated in strigolactone (SL) biosynthesis, suppress the irregular xylem associated phenotypes of Trichome Birefringence-Like 29 (TBL29), which is implicated in xylan O-acetylation. This previous work provided a functional link between SL biosynthesis/ signaling and secondary cell wall deposition. Here, the authors more broadly characterize the genetic relationships between multiple secondary cell wall mutants compromised in cellulose, hemicellulose and lignin biosynthesis and

multiple genes implicated in SL biosynthesis and signaling. The results suggest that SL biosynthesis is broadly required to mediate the irregular xylem syndrome of phenotypes, since max4 mutants broadly suppress each class of secondary cell wall biosynthesis mutant examined. The authors also suggest that these effects may be mediated independent of SL perception.
Overall, I think that this is a nice paper, and I have only a few comments that are meant to improve the quality of the paper.
Major criticisms
• Figure 5 is very important, as it suggests that tbl29 mutant suppression is independent of the canonical signaling perception pathway. It would be beneficial if the authors could include another figure panel describing the steps of SL biosynthesis and perception that are being tested in each of these double mutants to make it clearer to the reader in the figure why different results are being obtained, and why this might be interesting.

A new Figure 6 was included in the revised version of the manuscript following the suggestions made by Reviewer#2. Figure 6 summarizes the SL biosynthesis/perception pathway including the steps catalyzed by MAX1, MAX2, MAX3 and MAX4 (steps analyzed using our double mutant strategy). We also included the potential pathways connecting secondary wall defects to irregular xylem syndrome, according to our results.

Minor criticisms
• In the introduction, please change "which is then used as substrate to generate carlactone" to "which is then used as a substrate to generate carlactone".
The text was modified accordingly
